# Klotho-beta overexpression as a novel target for suppressing proliferation and fibroblast growth factor receptor-4 signaling in hepatocellular carcinoma

**DOI:** 10.1186/1476-4598-11-14

**Published:** 2012-03-23

**Authors:** Weijie Poh, Winnie Wong, Huimin Ong, Myat Oo Aung, Seng Gee Lim, Boon Tin Chua, Han Kiat Ho

**Affiliations:** 1 Institute of Medical Biology, A∗STAR (Agency for Science, Technology and Research), 8A Biomedical Grove, Immunos, Singapore 138648, Republic of Singapore; 2 Department of Pharmacy, National University of Singapore, 18 Science Drive 4, Singapore 117543, Singapore; 3 Departments of Gastroenterology and Hepatology, National University Hospital, Singapore 119074, Republic of Singapore

**Keywords:** FGFR4, Hepatocellular carcinoma, KLB, Liver stemness

## Abstract

**Background:**

We had previously demonstrated overexpression of fibroblast growth factor receptor-4 (FGFR4) in hepatocellular carcinoma (HCC). However, additional molecular mechanisms resulting in amplified FGFR4 signaling in HCC remain under-studied. Here, we studied the mechanistic role of its co-receptor klotho-beta (KLB) in driving elevated FGFR4 activity in HCC progression.

**Results:**

Quantitative real-time PCR analysis identified frequent elevation of KLB gene expression in HCC tumors relative to matched non-tumor tissue, with a more than two-fold increase correlating with development of multiple tumors in patients. KLB-silencing in Huh7 cells decreased cell proliferation and suppressed FGFR4 downstream signaling. While transient repression of KLB-FGFR4 signaling decreased protein expression of alpha-fetoprotein (AFP), a HCC diagnostic marker, prolonged inhibition enriched for resistant HCC cells exhibiting increased liver stemness.

**Conclusions:**

Elevated KLB expression in HCC tissues provides further credence to the oncogenic role of increased FGFR4 signaling in HCC progression and represents a novel biomarker to identify additional patients amenable to anti-FGFR4 therapy. The restricted tissue expression profile of KLB, together with the anti-proliferative effect observed with KLB-silencing, also qualifies it as a specific and potent therapeutic target for HCC patients. The enrichment of a liver stem cell-like population in response to extended KLB-FGFR4 repression necessitates further investigation to target the development of drug resistance.

## Introduction

With approximately 680,000 deaths in 2008, hepatocellular carcinoma (HCC) ranks third worldwide in cancer-related mortalities [1]. The majority of HCC patients present at advanced stages where curative surgical treatments are not applicable and face median survival of less than a year [2]. Moreover, traditional systemic chemotherapies have produced no significant survival benefit in advanced HCC patients [3]. Consequently, novel therapies for unresectable HCC are urgently needed to address this grim prognosis.

Recently, there has been increasing interest in developing molecular-targeted therapies that can discriminate between cancer cells and their non-neoplastic counterparts by targeting proteins overexpressed in tumors. The approval of sorafenib, a multi-targeted tyrosine kinase inhibitor, for the treatment of advanced-stage HCC patients in 2007 highlights the potential of such therapies in treating this complex neoplasia [4]. Furthermore, the success of sorafenib has intensified efforts in identifying receptor tyrosine kinase (RTK) genes frequently and selectively upregulated in HCC [5,6].

One promising RTK candidate in HCC is the fibroblast growth factor receptor-4 (FGFR4). Within the FGFR family, FGFR4 is the dominant isoform expressed in mature hepatocytes and is expressed at higher levels in the liver compared to other organs [7,8]. Amplified FGFR4 signaling has been reported in head-and-neck squamous cell carcinoma [9], and has also been linked to accelerated tumor progression in breast and colon cancers [10]. Transgenic mouse models demonstrated that ectopic expression of FGF19, a FGFR4-specific ligand, promotes hepatocyte proliferation and spontaneous HCC development [11]. In agreement with these studies, our group previously found elevated FGFR4 expression in HCC tumors and demonstrated that dysregulated FGFR4 signaling promotes proliferation [12]. These findings strongly implicate abnormal FGFR4 activity in HCC progression.

A key regulator of the FGFR4 pathway is its co-receptor klotho-beta (KLB), a 130 kDa trans-membrane protein that exhibits a more restricted expression profile in adipose, liver and pancreas tissues [8]. KLB and FGFR4 are both expressed at high levels in mature hepatocytes [8], where KLB stabilizes FGF19-FGFR4 binding to regulate production of cholesterol 7α-hydroxylase (CYP7A1) and hepatocyte proliferation [8,11,13]. Also, KLB^-/- ^and FGFR4^-/- ^knockout mice exhibit similar features such as increased CYP7A1 expression and small gallbladder size, demonstrating an intimate role of KLB in mediating FGFR4 activity in hepatocytes [14-16]. Given the contributory role of FGFR4 in HCC progression, we speculate that dysregulated KLB expression in HCC may function as an additional mechanism resulting in amplified FGFR4 signaling. While previous studies on KLB in hepatocytes focused mainly on its metabolic functions [8,13,17], this is the first study investigating its role in HCC progression.

We detected elevated KLB expression in HCC tumors that correlated with multi-foci formation. Repression of KLB expression suppressed FGFR4 downstream signaling and significantly inhibited hepatoma cell proliferation. Intriguingly, we observed biomarkers suggestive of an elevated liver stemness behavior in Huh7 hepatoma cells after prolonged repression of KLB-FGFR4 signaling. The discovery of amplified KLB expression in HCC expands upon our previous work demonstrating dysregulated FGFR4 signaling in HCC progression. The enrichment of cells expressing stemness markers in response to FGFR4 inhibition highlights the need to characterize this stem-like population and elucidate the underlying resistance mechanisms. This information will be instrumental in identifying the repertoire of HCC-specific pathways to be targeted concurrently with FGFR4 signaling to achieve effective treatment.

## Materials and methods

### Reagents

FGFR inhibitor PD173074 (Calbiochem, San Diego, CA) was re-suspended in DMSO and stored at -20°C.

### Cell culture

HepG2, Sk-Hep1, Hep3B, PLC/PRF/5, Hs817.T, THLE2 were obtained from ATCC (Manassas, VA) while Huh7 was obtained from the Max Planck Institute (Martinsried, Germany). HepG2, Hep3B, PLC/PRF/5 cells were maintained in MEM supplemented with 10% FBS, sodium pyruvate and non-essential amino acids, THLE2 in BEGM (Lonza, Basel, Switzerland) and the rest in DMEM with 10% FBS and sodium pyruvate. Huh7 cells were cultured for eight weeks in PD173074 (0.1 μM - 1.0 μM) to generate resistant HCC cells. Cells were maintained at 37°C in 5% CO_2_. Unless stated otherwise, reagents were obtained from Invitrogen (Life Technologies, Grand Island, NY).

### HCC patient sample preparation

Total RNA was isolated from 56 paired tumors and adjacent non-tumor liver tissues from HCC patients at the National University Hospital (Singapore) as previously described in accordance with ethics guidelines [12]. The National Healthcare Group Institutional Review Board approved this study. cDNAs were synthesized using SuperScript III reverse transcriptase (Invitrogen, Carlsbad, CA) according to manufacturer's instructions.

### Gene expression analysis

Quantitative real-time polymerase chain reaction (qRT-PCR) was performed in triplicates on the ABI7500 Fast Real-Time PCR system (Applied Biosystems, Foster City, CA) with Power SYBR Green PCR Master Mix (ABI). The following gene-specific primer pairs were used:

KLB (NM_175737.3): (F) 5'-GCAGTCAGACCCAAGAAAATACAGA-3',

(R) 5'-CCCAGGAATATCAGTGGTTTCTTC-3';

AFP (NM_001134.1), (F) 5'-AGCTTGGTGGTGGATGAAAC-3',

(R) 5'-TCTTGCTTCATCGTTTGCAG-3';

CD133 (NG_011696.1), (F) 5'-CCTGGGGC-TGCTGTTTATTA-3',

(R) 5'-TACCTGGTGATTTGCCACAA-3',

CD44 (NG_008937.1), (F) 5'-CGGACACCATGGACAAGTTT-3',

(R) 5'-GAAAGCCTTGCAGAGGTCAG-3';

FGFR4 (NG_012067.1), (F) 5'-CAAAGACAACGCCTCTGACA-3',

(R) 5'- CACCAAGCAGGTTGATGATG-3';

GAPDH (NM_002046.3), (F) 5'-ATGTTCGTCATGGGTGTGAA-3',

(R) 5'-TGTGGTCATGAGTCCTTCCA-3'

Gene expression was analyzed with the 2^-ΔΔCt ^formula after normalizing to GAPDH.

### Liver tumor tissue lysates

Protein lysates of matched normal and tumor liver tissues were obtained from Protein Biotechnologies (Ramona, CA).

### Immunoblotting

Cells were lysed in buffer (50 mM HEPES, 150 mM NaCl, 1 mM EDTA, 10% glycerol, 1% TritonX-100, 10 mM sodium pyrophosphate) containing protease and phosphatase inhibitors (10 mM sodium fluoride, 2 mM sodium orthovanadate, 1 mM phenylmethylsulfonyl fluoride, 0.1 μg/mL aprotinin). Protein concentrations were determined by the BCA method (Pierce-Thermo Fisher Scientific, Rockford, IL) and equal amounts were resolved with 7.5% SDS-PAGE before electro-transferring to PVDF or nitrocellulose membranes. After blocking, blots were incubated in antibodies diluted in PBS with 0.05% (v/v) Tween 20 and 5% (w/v) non-fat milk or bovine serum albumin (BSA). Primary antibodies used were anti-proliferating cell nuclear antigen PCNA (P10) from Santa Cruz Biotechnology (Santa Cruz, CA), phospho-FRS2α, anti-phospho-p44/42 ERK1/2, anti-phospho-Akt antibodies from Cell Signaling Technology (Beverly, MA), anti-CD133 from Miltenyi Biotec GmbH (Bergisch Gladbach, Germany) and anti-β-tubulin 1 from Sigma (St Louis, MO). Blots were visualized after incubation with secondary anti-mouse and anti-rabbit HRP-conjugated antibodies (Pierce) by enhanced chemiluminescence (Pierce).

### Gene silencing by siRNA and shRNA

24 h after seeding (2 × 10^5 ^cells/well) in a six-well plate, cells were transfected with 100 nM non-targeting control siRNA (D-0018010-10), KLB-targeting (D-017848-01) or FGFR4-targeting (J-003134-15) siRNA from Dharmacon (Chicago, IL) according to manufacturer's instructions. Gene knockdown was confirmed by qRT-PCR 48 h post-transfection, and immunoblotting 72 h after transfection. To establish stable FGFR4 knockdown clones, Huh7 cells were transfected with pRS vector control (TR20003, Origene Technologies, Rockville, MD) and FGFR4-specific HuSH 29mer shRNA constructs (TR320356, Origene) and selected with 2 μg/mL puromycin (Sigma) for at least 14 days as previously described [12].

### Cell proliferation assay

48 h post-transfection with KLB-targeting siRNA, eight replicates (7500 cells/well) were seeded in a 96-well plate. After 24 h, CellTiter-Glo luminescent assay (Promega, Madison, WI) was performed to measure ATP content as a gauge of viable cells.

### Statistical analysis

All categorical data was tested with Fisher's exact probability test. Differences in transcript levels were analyzed using Student's *t *test of ΔCt values or one-way ANOVA and Tukey's multiple comparison tests. Comparisons of KLB mRNA, protein levels between paired tumor and normal tissues were carried out by non-parametric Wilcoxon matched paired test. All statistical analyses were performed using GraphPad Prism software (GraphPad Software Inc, San Diego, CA). Patient clinicopathological parameters, provided in Additional file 1: Table S1, including tumor multiplicity, age, gender, etiology, disease outcome, alanine aminotransferase (ALT), aspartate aminotransferase (AST), alpha-fetoprotein (AFP) levels, tumor histology and diameter, presence of cirrhosis and vascular invasion were evaluated. Statistical significance is defined as *P *< 0.05.

## Results

### KLB expression is frequently upregulated in HCC

**Figure 1:**
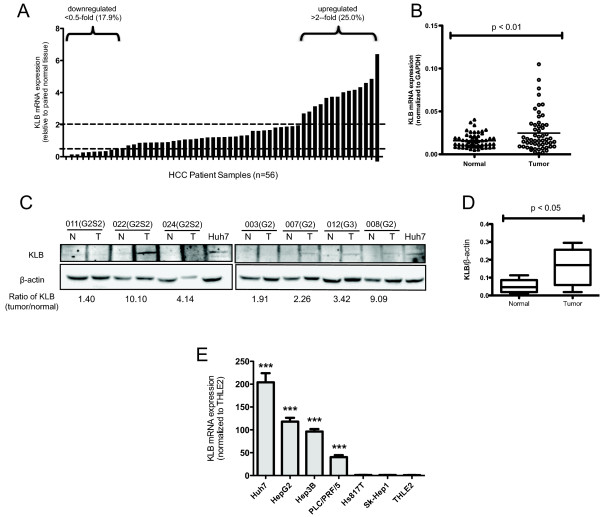
**KLB expression is upregulated in HCC tumors and cell lines**. (A) Analysis of KLB gene expression using qRT-PCR in tumor tissue relative to its matched normal tissue after normalizing to GPADH mRNA. Fold change of < 0.5 indicates down-regulation of KLB expression while > 2-fold change indicates KLB up-regulation in tumors. (B) Wilcoxon signed rank test was used to analyze the difference of KLB gene expression in paired normal (Δ) and tumor liver tissues (○). (C) KLB protein expression was determined in Huh7 cells and an independent set of paired HCC lysates. The ratio of KLB between tumor and normal tissue was calculated as (tumor KLB/normal KLB) after normalizing the protein band density to β-actin. (D) The Wilcoxon signed rank test was used to analyze the difference in KLB/β-actin expression between the two groups. (E) KLB mRNA expression in HCC cell lines are expressed as fold changes relative to THLE2 with error bars representing standard deviation (SD) converted to fold changes. (***, *P *< 0.001 using Student's *t*-test).

Using qRT-PCR, we studied KLB mRNA levels in 56 human HCC tumors and their adjacent paired normal liver tissues to identify expression level changes that accompany cancer progression (Figure 1A, Table 1). Upregulated KLB expression (> 2-fold) in tumor tissue was observed in 25% of HCC samples and paired *t-test *analysis indicates an overall statistically significant increase in KLB expression in HCC tumors (Wilcoxon matched pairs test, *P *< 0.01, Figure 1B). We found no significant correlation of KLB gene expression with FGFR4 previously determined from the same patient population [12] (Spearman rank correlation: *r *= 0.032; *P *= 0.812). We also performed *in silico *analysis of published microarray data from Oncomine http://www.oncomine.org that revealed KLB overexpression in tumors relative to adjacent normal tissues in HCC patients and also livers from normal patients (Additional file 2: Figure S1). As shown in Figure 1C, analysis of KLB protein expression in an independent set of paired HCC liver lysates revealed KLB up-regulation in HCC tumors relative to adjacent normal tissues, which concurs with our qRT-PCR and *in silico *results. Protein levels are statistically different between HCC tumors and paired normal tissues (Wilcoxon matched pairs test, *P *< 0.05, Figure 1D right panel).

**Table 1:** Correlation of KLB expression and tumor multiplicity in HCC patients

	Down-regulated KLB expression (× < 0.5-fold)	mRNA change not significant (2.0 > × > 0.5)	Up-regulated KLB expression (× > 2.0)	p-value
	**N = 10 (17.86%)**	**N = 32 (57.14%)**	**N = 14 (25%)**	
				
**Tumor multiplicity:**				
				
Single tumor	**8**	**22**	**4**	**0.02**
Multiple tumors	**2**	**10**	**10**	
				
**Median age**	**53.5**	**59.5**	**55.3**	**0.23**
				
**Gender:**				
				
Female	**1**	**7**	**3**	**0.81**
Male	**9**	**25**	**11**	
				
**Etiology**				
				
HBV	**7**	**23**	**11**	**1.00**
HCV	**0**	**2**	**1**	
Alcohol	**0**	**1**	**0**	
				
**Outcome**				
				
Alive	**3**	**11**	**4**	**0.93**
Death	**4**	**10**	**3**	
Recurrence	**1**	**6**	**3**	
				
**Mean ALT (U/ml)**	**47.8**	**65.3**	**48.5**	**0.58**
				
**Mean AST (U/ml)**	**63.8**	**70.6**	**67**	**0.95**
				
**Mean AFP (U/ml)**	**45550**	**19734**	**14240**	**0.43**
				
**Tumor histology:**				
				
Poor	**5**	**9**	**1**	**0.72**
Well	**1**	**5**	**0**	
				
**Mean diameter**	**10.03**	**8.35**	**9.32**	**0.64**
				
**Cirrhosis:**				
				
Positive	**7**	**19**	**11**	**0.43**
Negative	**3**	**13**	**3**	
				
**Vascular Invasion:**				
				
Yes	**7**	**16**	**7**	**0.52**
No	**2**	**13**	**4**	

Using the classification in Figure 1 to stratify patients based on KLB expression in HCC tumors relative to their respective normal tissues, Fisher's exact test was performed with respect to clinicopathological features.

### Increased KLB expression associates with the development of multiple HCC tumors

To determine the clinical relevance of elevated KLB expression in HCC patients, we correlated the clinicopathological features between patients with upregulated (> 2-fold) and down-regulated (0.5-fold) KLB mRNA in the respective HCC tissues. Fisher's exact test results indicate that KLB overexpression significantly correlated with multi-foci manifestation in patients (*P *< 0.05, Table 1). There was no significant correlation with other clinicopathological parameters.

### HCC cell lines show KLB gene overexpression

We next evaluated KLB mRNA expression in six HCC cell lines and a normal liver cell line THLE2. In agreement with our clinical findings, KLB was overexpressed in four HCC cell lines (Huh7, HepG2, Hep3B, PLC/PRF/5) (Figure 1E). SK-Hep1 was shown to be of endothelial origin lacking hepatic-specific features, which may explain the lack of concordance with other HCC cell lines [18]. We selected Huh7, which exhibited the highest KLB gene expression, to study the contribution of KLB to FGFR4 signaling and HCC progression. Moreover, we previously found a homozygous FGFR4 G388R polymorphism in Huh7 that is linked to increased HCC aggressiveness [12]. Hence Huh7 provides a good culture model to study the effects of KLB perturbation in a more aggressive setting.

### Silencing KLB expression decreases HCC cell growth via cell cycle arrest

**Figure 2:**
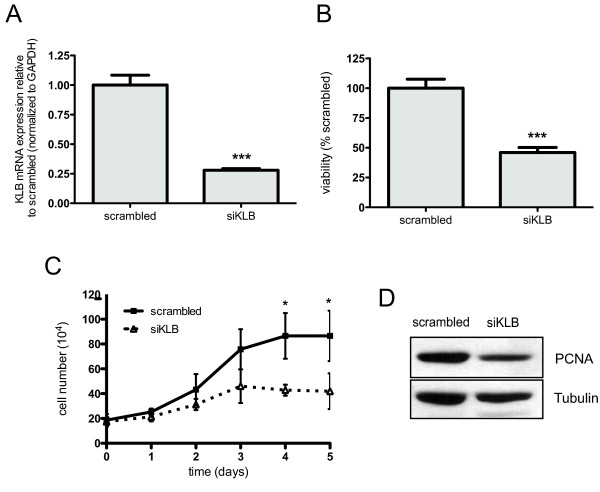
**Silencing of KLB with siRNA suppresses cell viability and proliferation**. Huh7 cells were transfected with 100 nM scrambled control or KLB-targeting siRNA. (A) After 48 h, qRT-PCR was performed to confirm KLB knockdown after normalizing to GAPDH. Error bars represent SD converted to fold changes. (B) At 48 h post transfection, Huh7 cells were re-seeded in a 96-well plate. After additional 24 h incubation, cell viability was measured using CellTiter-Glo and evaluated relative to cells transfected with scrambled siRNA. (C) At the indicated time points after transfection, Huh7 cells were harvested for counting. (D) Immunoblotting with PCNA antibody was performed 72 h post siRNA transfection using tubulin as loading control. For A-C, means of three independent experiments are indicated with SD as error bars. (***, *P *< 0.001; *, *P *< 0.05 using Student's *t*-test).

**Figure 3:**
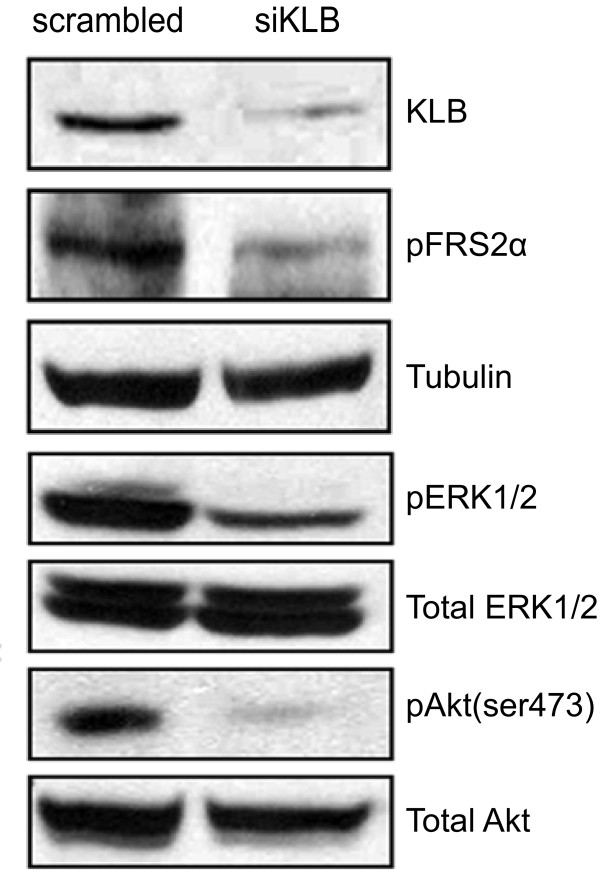
**KLB mediates FGFR4 downstream signaling and phosphorylation of pro-survival proteins**. 72 h post-transfection with siRNA, Huh7 cells were lysed, resolved with 7.5% SDS-PAGE and immunoblotted using the indicated antibodies. Results are indicative of two independent experiments.

Having noted elevated KLB expression in HCC patients and cell lines, we evaluated the effect of RNAi targeting KLB on cell growth in Huh7. We confirmed effective silencing at the transcript (Figure 2A) and protein levels (Figure 3) in cells transfected with KLB-targeting siRNA. Since KLB can also stimulate activation of FGFR1c, 2c, 3c and 4 [13], we selected CYP7A1, a physiological target specifically repressed by FGFR4 in hepatocytes [19], as a marker of FGFR4 activity. We validated KLB-silencing on FGFR4 signaling by showing that CYP7A1 was upregulated by more than 3-fold (Additional file 3: Figure S2). More importantly, KLB-silenced cells displayed more than 50% reduction in proliferation compared to cells transfected with control siRNA (Figure 2B). Next, we analyzed the growth curve of Huh7 cells and observed statistically significant growth suppression in cells transfected with KLB-targeting siRNA at day 4 and 5 post-transfection (*P *< 0.05, Figure 2C). Decreased PCNA protein expression suggests that growth reduction is due to cell cycle arrest in these cells (Figure 2D).

### Decreased KLB expression inhibits FGFR4 downstream signaling

Activated FGFRs phosphorylate the docking protein FGF receptor substrate-2α (FRS2α), which then activates the Ras-Raf-ERK1/2 MAPK and PI-3 kinase pathways responsible for cell proliferation and survival. Huh7 cells express high levels of FGFR4 and exhibit significant basal phosphorylation of FRS2α in the absence of FGF19 stimulation, hence this cell model allows us to study the effects of KLB suppression independent of the influence of FGF19 [12]. At 72 h post siRNA transfection, we observed decreased KLB protein expression with a corresponding reduction in phosphorylated FRS2α, ERK1/2 and Akt proteins, indicative of decreased FGFR4 signaling (Figure 3).

### siRNA-mediated silencing of KLB decreases AFP protein levels with an increase in transcript levels of AFP and stemness associated genes CD133, CD44

**Figure 4:**
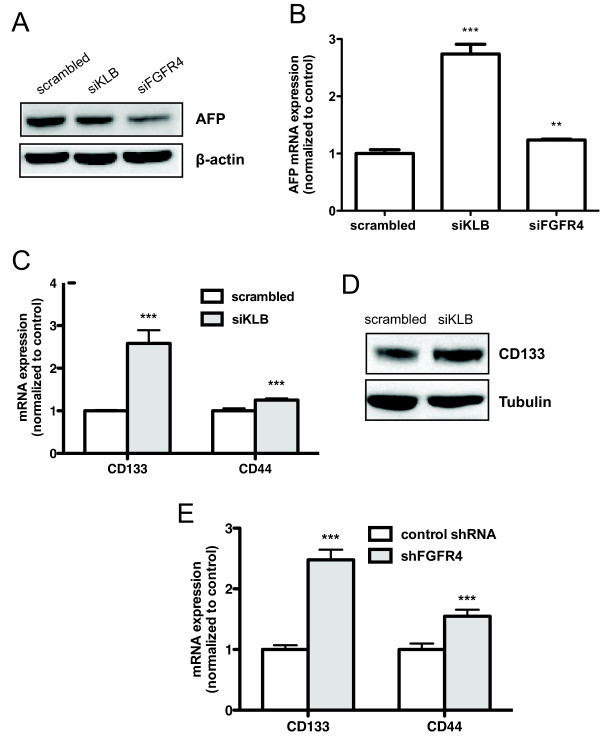
**Silencing of KLB or FGFR4 increases expression of liver stemness genes**. (A) 48 h post siRNA silencing of KLB or FGFR4 in Huh7 cells, AFP gene expression was determined by qRT-PCR. Data indicates fold change in gene expression normalized to scrambled siRNA. Error bars represent SD converted to fold changes. (B) After 72 h, AFP protein expression was also determined using polyclonal anti-AFP antibodies, normalized to β-actin as loading control. (C) CD133 and CD44 expression after KLB-silencing was subsequently determined as described for (A). (D) The effect of the silencing on CD133 protein expression was performed using anti-CD133 antibody. (E) CD133 and CD44 mRNA levels were measured in Huh7 stable clones expressing control shRNA and FGFR4 shRNA after normalizing to GAPDH.

Next, we monitored the protein levels of AFP, a clinically relevant HCC biomarker, in Huh7 cells after siRNA-mediated silencing of either KLB or FGFR4. In KLB- or FGFR4-silenced cells, AFP protein expression decreased (Figure 4A), mirroring previous work showing suppressed AFP secretion with inhibition of FGFR4 signaling [12]. The weaker suppression on AFP protein expression in KLB-silenced cells compared to cells transfected with FGFR4-targeting siRNA could be due to a compensatory elevation of FGFR4 expression with KLB suppression (Additional file 4: Figure S3). Intriguingly, we observed an increase of 170% and 20% in AFP mRNA expression in KLB- and FGFR4-silenced cells respectively. To investigate the opposing effects that inhibiting FGFR4 signaling has on AFP protein and transcript levels, we considered the expression of AFP in hepatic progenitor cells and its role in development of stem-like features in HCC [20,21]. We postulated that RNAi-mediated silencing of KLB or FGFR4 eliminated the bulk hepatoma population exhibiting hyper-activated FGFR4 signaling and AFP secretion, thereby resulting in an overall decrease in AFP protein levels shown in Figure 4A. However, this strategy inadvertently enriched for resistant cells with increased stemness behavior that contributed to elevated AFP gene expression. To circumvent the dichotomy associated with AFP, we decided to focus on CD133 and CD44, bona fide stemness-associated genes in liver progenitor cells [22-24]. We observed a significant increase of 150% and 25% in the expression of CD133 and CD44 genes respectively in KLB-silenced Huh7 cells, strongly indicating an elevated response in liver stemness gene expression (Figure 4C). CD133 protein expression also confirmed that KLB suppression enriched for cells with higher CD133 expression (Figure 4D).

### Stable repression of FGFR4 pathway increases CD133, CD44 gene expression

Since KLB is a key regulator of the FGFR4 pathway in hepatocytes, we examined CD133 and CD44 mRNA expression in Huh7 cells with stables repression of FGFR4 characterized previously [12]. qRT-PCR analysis indicated a significant increase of 150% and 50% in CD133 and CD44 expression in cells stably transfected with FGFR4 shRNA compared to Huh7 cells expressing control shRNA (Figure 4E).

### Sustained exposure to sub-lethal concentrations of PD173074, a FGFR inhibitor, selected for resistant cells exhibiting a dose-dependent increase in CD133 and CD44 expression

**Figure 5:**
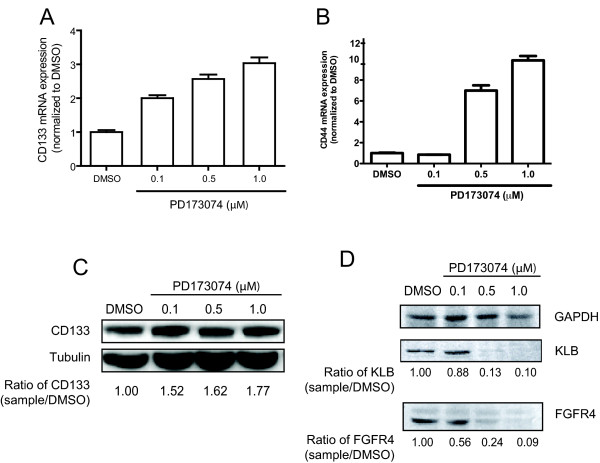
**CD133 and CD44 expression are upregulated in PD173074-resistant cells**. Resistant Huh7 cells were generated by culturing in sub-lethal concentrations of PD173074 (0.1 μM - 1.0 μM). After eight weeks, (A) CD133 and (B) CD44 mRNA expression were analyzed by qRT-PCR. Results are indicated with SD converted to fold changes as error bars. The differences were statistically significant (*P *< 0.001 by one-way ANOVA, *P *< 0.05 by Tukey's multiple comparison test). (C) CD133 protein expression was determined by immunoblotting and normalized to β-actin. (D) Long term effect of PD173074 treatment on FGFR4 and KLB protein expression were determined by immunoblot assays. For (C) and (D), the respective relative protein levels were calculated relative to DMSO control after normalizing the protein band density to that of the loading control.

Previously, we showed that PD173074, a non-selective FGFR small molecule inhibitor, suppresses viability and invasiveness more potently in HCC cells with high endogenous FGFR4 expression [12]. Here, we determined if the increased stemness behavior with transient and prolonged inhibition of FGFR4 signaling could be observed with PD173074 treatment. We cultured Huh7 cells for eight weeks under sub-lethal concentrations of PD173074 (0.1 μM - 1.0 μM) and observed a dose-dependent increase in CD133 (Figure 5A) gene expression in PD-resistant Huh7 cells. A similar increase in CD44 gene expression with higher doses was also noted, with the exception at the lowest 0.1 μM dose (Figure 5B). CD133 protein expression was increased in the resistant population consistent with the qRT-PCR results (Figure 5C). FGFR4 and KLB protein levels in PD-resistant cells were also significantly suppressed in a dose-dependent manner (Figure 5D).

## Discussion

HCC is a solid tumor lacking a dominant pathogenetic event driving cancer development and progression. The molecular complexity of this cancer warrants a multi-disciplinary approach targeting multiple dysregulated signaling pathways [5]. Aberrant activation of RTK signaling has been observed in HCC and the clinical benefits observed with sorafenib further support the efficacy of targeting multiple RTK pathways in this disease [5,6]. As such, establishing a repertoire of pathways frequently and selectively upregulated in tumor cells will facilitate the development of more specific anti-cancer therapeutics with minimal toxicity to non-malignant counterparts.

Our lab has previously showed that frequent elevation of FGFR4 expression occurs in HCC tumors and is linked to increased proliferation and aggressiveness [12]. Yet, the understanding of FGFR4 signaling on hepatocarcinogenesis remains divided with counter-evidence suggesting that removal of FGFR4 delays diethylnitrosamine-induced HCC formation [25-27]. This dichotomy is likely marred by the complex interplay with other tumor initiating factors in animal model systems, hence we decided to base our study in a more relevant setting using human paired tumor/normal samples. To uncover additional mechanisms amplifying FGFR4 activity in HCC, we examined its co-receptor KLB which is required for ligand binding and activation of FGFR4 signaling in hepatocytes [8]. While others have investigated the impact of KLB-FGFR4 signaling on bile acid metabolic pathways in hepatocytes [8,13,15], details concerning aberrations of KLB in HCC development are lacking.

In this study, we observed frequent elevation of KLB expression in HCC tumors relative to paired normal liver tissues and noted that KLB up-regulation correlated with multiple tumor formation. We found a similar increase in KLB mRNA expression in HCC cell lines as well as in published microarray data. Next, we confirmed increased KLB protein expression in an independently obtained panel of HCC paired lysates. Taken together, our findings suggest that increased KLB expression is a frequent event in normal-to-tumor transition in hepatocytes. Although KLB has also been known to activate FGFR1c, 2c, and 3c, there is a lack of published evidence suggesting that activation of these FGFRs has any pro-tumorigenic effect in hepatocytes [13]. On the other hand, FGFR4 has been proposed as a novel RTK target for HCC based on studies where FGF19-mediated FGFR4 activation resulted in hepatocyte proliferation and HCC formation [11,28]. In this study, we built upon our previous work by focusing on FGFR4 signaling and have uncovered a novel molecular mechanism amplifying FGFR4 activity, further corroborating a tumor-progressive role of dysregulated FGFR4 activity in HCC.

To elucidate a role for KLB over-expression in HCC tumors, we silenced KLB expression in Huh7 that has the highest KLB level in our HCC cell line panel. Moreover, we previously reported that Huh7 cells are homozygous for the FGFR4 G388R polymorphism linked to increased aggressiveness [12]. Hence, we are able to examine the effects of KLB perturbation in an aggressive hepatoma model driven by elevated FGFR4 activity. We observed a significant reduction in proliferation due to cell cycle arrest and suppressed phosphorylation of FRS2α, ERK1/2 and Akt with KLB-silencing. In cancer cells, ERK1/2 activation increases expression of genes promoting cell cycle progression while phosphorylated Akt inhibits apoptosis [29,30]. Given the poor prognosis in HCC patients exhibiting increased activation of ERK1/2 and Akt, both pathways are attractive targets to disrupt HCC progression [31]. Moreover, protein expression of AFP, a HCC diagnostic marker, was repressed when either FGFR4 or KLB was silenced, which is consistent with our earlier finding where inhibition of FGFR4 activity with PD173074 or RNAi silencing of FGFR4 blocked AFP secretion [12]. Together, these results underline a potent anti-tumor effect of KLB inhibition in HCC. Importantly, the normal phenotype of KLB null mice, coupled with the restricted tissue expression of KLB [14-16], suggests that systemic treatment with KLB inhibitors should have minimal risk of toxic effects in HCC patients.

Given the paradoxical increase in liver tumor marker AFP transcription when either FGFR4 or KLB was silenced, we considered AFP's role in cellular de-differentiation and stem-like behavior in HCC [21]. We propose that the inhibition of FGFR4 signaling through KLB-silencing in HCC cells eliminated the hyper-proliferative FGFR4-overexpressing cell population, while enriching for resistant cells with progenitor features. The disparity between the transcript and protein expression could be a temporal phenomenon due to the inhibitory effect of suppressing FGFR4 signaling on AFP production with the simultaneous emergence of a stem-like population. Hence, we used established stem-cell markers CD133 and CD44 to avoid the confounding effects associated with AFP analysis. CD133 and CD44 are cell surface markers that have been used to isolate progenitor cell sub-populations from breast, colon and liver tumors that exhibit increased metastatic potential, chemoresistance and tumorigenicity [22-24,32]. In agreement with our finding, it has been reported that CD133^+ ^cells are enriched in liver cancer cell lines treated with traditional chemotherapy drugs doxorubicin and fluorouracil [32]. However, other work suggests that CD133 may also be expressed in differentiated tumor cells [33]. Therefore, we envisage a combination of CD133 and CD44 as a more reliable characterization of stem-like character, consistent with a notable report proposing CD133^+ ^CD44^+ ^cells isolated from Huh7 as bona fide cancer progenitor cells [24]. We found up-regulation of CD133 and CD44 genes in KLB-silenced Huh7 cells and in clones with stable repression of FGFR4 shRNA. Similarly, we observed a dose-dependent increase in CD133 and CD44 expression in resistant Huh7 clones cultured under sub-lethal concentrations of FGFR inhibitor PD173074. Overall, our work supports the notion that while inhibition of FGFR4 signaling exerts acute and direct anti-proliferative effects on HCC cells, the surviving sub-population may be clonally selected for a resilient, stem cell-like phenotype. Moving forward, analyzing the expression of other putative hepatic stem cell markers and *in vivo *transplantation studies of these stem-like subpopulations will benefit our understanding of the underlying molecular characteristics imparting resistance.

In conclusion, elevated KLB expression in HCC tumors contributes to malignant progression and represents an additional mechanism of aberrant FGFR4 signaling in HCC. This result supports our previous finding highlighting the oncogenic role of FGFR4 signaling in HCC. The lack of overlap in KLB and FGFR4 overexpression in HCC tumors points to a larger population of patients exhibiting elevated FGFR4 signaling, affirming the need for FGFR4 pathway inhibitors. In this study, the anti-proliferative outcome of repressing KLB expression, coupled with its restricted tissue distribution, strongly points to KLB as a potent, more selective and novel target for therapeutic intervention of aberrant FGFR4 signaling in HCCs. In view of the increased stemness gene expression observed with repression of FGFR4 signaling, clinical development of FGFR4 inhibitors will benefit from complementary therapeutics that target the resistant liver stem cell-like population concurrently to achieve superior outcome in HCC patients.

## Abbreviations

AFP: Alpha-fetoprotein; FGF19: Fibroblast growth factor 19; FGFR4: Fibroblast growth factor receptor-4; FRS2α: FGF receptor substrate protein-2α; GAPDH: Glyceraldehyde 3- phosphate dehydrogenase; HCC: Hepatocellular carcinoma; KLB: Klotho-beta; PCNA: Proliferating cell nuclear antigen; siRNA: Small interfering ribonucleic acid; shRNA: Short hairpin ribonucleic acid; RTK: Receptor tyrosine kinase.

## Competing interests

The authors declare that they have no competing interests.

## Authors' contributions

WP carried out the molecular genetic studies and drafted the manuscript. WW and HO assisted with *in vitro *experiments and data analysis. MO and SL processed the patient liver samples and collected clinical information. HH and BC conceived of the study, participated in its design and coordination and helped to draft the manuscript. All authors read and approved the final manuscript.

## Supplementary Material

Additional file 1 **Table S1 Clinicopathological parameters of HCC patients**. Click here for file

Additional file 2 **Figure S1 KLB mRNA is overexpressed in HCC (Oncomine expression array data)**. Expression of KLB mRNA in HCC tissue relative to (A) adjacent non-tumor liver tissues [34] and (B) normal liver tissues from normal patients [35]. Click here for file

Additional file 3 **Figure S2 Upregulation of CYP7A1 mRNA in KLB-silenced Huh7 cells**. 48 h post siRNA transfection in Huh7 cells, CYP7A1 gene expression was measured by qRT-PCR and normalized to GAPDH. Results are indicated with SD converted to fold changes as error bars. Click here for file

Additional file 4 **Figure S3 Decreased KLB and FGFR4 expression with RNAi silencing**. 48 h post siRNA silencing of (A) KLB and (B) FGFR4 in Huh7 cells, gene expression of KLB and FGFR4 were measured by qRT-PCR and normalized to GAPDH. Results are indicated with SD converted to fold changes as error bars. Click here for file
