# Supplementary material for: Klotho-beta overexpression as a novel target for suppressing proliferation and fibroblast growth factor receptor-4 signaling in hepatocellular carcinoma
Source: Mol Cancer. 2012 Mar 23;11:14. doi: 10.1186/1476-4598-11-14 (PMC3361496; doi:10.1186/1476-4598-11-14)
Supplement: Additional file 1 — Table S1 Clinicopathological parameters of HCC patients. [file 1476-4598-11-14-S1.DOC]

Table S1. Clinicopathological parameters of HCC patients
