# Supplementary material for: Klotho-beta overexpression as a novel target for suppressing proliferation and fibroblast growth factor receptor-4 signaling in hepatocellular carcinoma
Source: Mol Cancer. 2012 Mar 23;11:14. doi: 10.1186/1476-4598-11-14 (PMC3361496; doi:10.1186/1476-4598-11-14)
Supplement: Additional file 2 — Figure S1 KLB mRNA is overexpressed in HCC (Oncomine expression array data). Expression of KLB mRNA in HCC tissue relative to (A) adjacent non-tumor liver tissues [34] and (B) normal liver tissues from normal patients [35]. [file 1476-4598-11-14-S2.DOC]

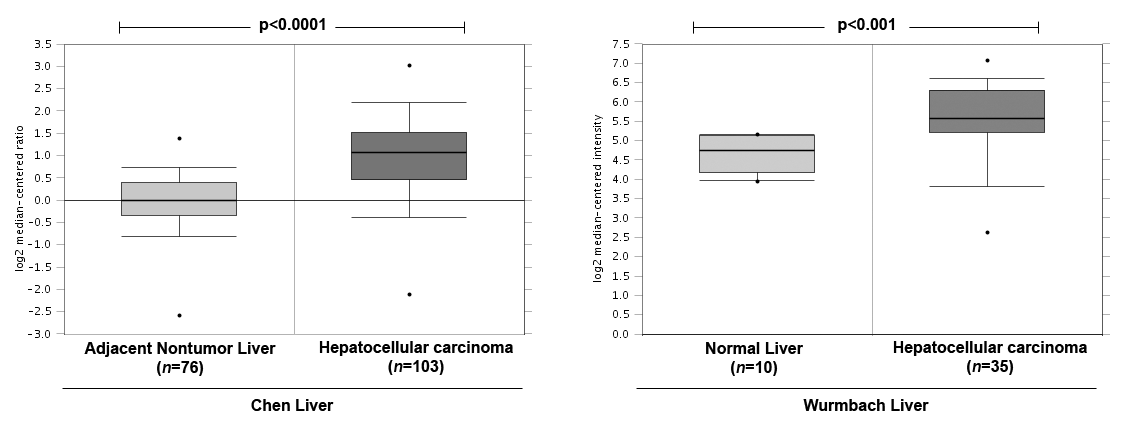


**Figure S1. KLB mRNA is overexpressed in HCC (Oncomine expression array data).** Expression of KLB mRNA in HCC tissue relative to (A) adjacent non-tumor liver tissues and (B) normal liver tissues from normal patients .
