# Supplementary material for: Klotho-beta overexpression as a novel target for suppressing proliferation and fibroblast growth factor receptor-4 signaling in hepatocellular carcinoma
Source: Mol Cancer. 2012 Mar 23;11:14. doi: 10.1186/1476-4598-11-14 (PMC3361496; doi:10.1186/1476-4598-11-14)
Supplement: Additional file 3 — Figure S2 Upregulation of CYP7A1 mRNA in KLB-silenced Huh7 cells. 48 h post siRNA transfection in Huh7 cells, CYP7A1 gene expression was measured by qRT-PCR and normalized to GAPDH. Results are indicated with SD converted to fold changes as error bars. [file 1476-4598-11-14-S3.DOC]

**Figure S2. Decreased KLB and FGFR4 expression with RNAi silencing.** 48 h post siRNA silencing of (A) *klb* and (B) *fgfr4* in Huh7 cells, gene expression of KLB and FGFR4 were measured by qRT-PCR and normalized to *gapdh*. Results are indicated with SD converted to fold changes as error bars.
